# Problem gamblers spend less money when loot boxes are removed from a game: a before and after study of *Heroes of the Storm*

**DOI:** 10.7717/peerj.7700

**Published:** 2019-10-29

**Authors:** David Zendle

**Affiliations:** Computer Science, University of York, York, United Kingdom

**Keywords:** Loot boxes, Gambling, Video game effects, Gaming, Media effects

## Abstract

Loot boxes are items in video games that may be paid for with real-world money, but which contain randomised contents. There is a reliable correlation between loot box spending and problem gambling severity: the more money gamers spend on loot boxes, the more severe their problem gambling tends to be. However, it is unclear whether this link represents a case in which loot box spending causes problem gambling; a case in which the gambling-like nature of loot boxes cause problem gamblers to spend more money; or whether it simply represents a case in which there is a general dysregulation in in-game spending amongst problem gamblers, nonspecific to loot boxes. The multiplayer video game *Heroes of the Storm* recently removed loot boxes. In order to better understand links between loot boxes and problem gambling, we conducted an analysis of players of *Heroes of the Storm* (*n* = 112) both before and after the removal of loot boxes. There were a complex pattern of results. In general, when loot boxes were removed from *Heroes of the Storm*, problem gamblers appeared to spend significantly less money in-game in contrast to other groups. These results suggest that the presence of loot boxes in a game may lead to problem gamblers spending more money in-game. It therefore seems possible that links between loot box spending and problem gambling are not due to a general dysregulation in in-game spending amongst problem gamblers, but rather are to do with specific features of loot boxes themselves.

## Introduction

*Heroes of the Storm* is a team-based multiplayer online video game. The game itself is free to play, and until 24th March 2019, *Heroes of the Storm* adopted a monetization strategy based around two different kinds of microtransactions. Under this strategy, players could spend real-world money on two different kinds of items: (1) they could directly purchase specific cosmetic upgrades for their characters via a premium currency; (2) they could pay real-world money via the same premium currency to buy loot boxes (known in-game as ‘loot chests’) which contained a randomised selection of cosmetic items.

After 24th March 2019, loot boxes were no longer available for purchase in *Heroes of the Storm*: They could still be obtained in-game by completing specific objectives but could no longer be bought via a premium currency. No official justification for the removal of loot boxes from the game was given. However, media sources commonly speculated that their removal was due to widespread concern regarding links between loot boxes and gambling (Boxer, 2019; Kaser, 2019).

These concerns centre around distinctive formal similarities between loot boxes and gambling. In Griffiths (1995), Griffiths specifies several characteristics that distinguish gambling from other activities: For example, when gambling, an unknown future event typically determines the exchange of money or valuable goods, with the involvement of chance. As noted in Drummond & Sauer (2018), this description fits some loot boxes just as well as it fits conventional forms of gambling. Connected to these arguments is the idea of a variable schedule of reinforcement: A pattern of rewards in which a behaviour is rewarded (or ‘reinforced’) after it has been engaged in a variable number of times (Skinner & Ferster, 2015). These intermittent patterns of reward are known to lead to continued engagement in a behaviour (Eysenck, 2004), and have been suggested as one of the drivers of problematic gambling (Blaszczynski & Nower, 2002). In particular, the implementation of such schedules in machine gambling has been repeatedly cited as key to the ability of these machines to engage gamblers (Griffiths, 1993; Walker et al., 2004). Both when opening loot boxes and when gambling, individuals stake something of real-world value on the chance outcome of an uncertain future event, in the hope of receiving something of greater value. Indeed, some loot boxes (such as in the first-person shooter game *Counter-Strike: Global Offensive*) are explicitly presented to gamers as a gambling-like spinning reel of prizes. Just as with a slot machine or other electronic gambling machine, such loot boxes provide an intermittent pattern of rewards, in which paying for a loot box is only rewarded after a variable number of attempts. Because of structural similarities structural similarities between gambling and gaming, researchers have speculated that some loot boxes may be “psychologically akin” (Drummond & Sauer, 2018) to gambling, and hence create a gateway to problem gambling amongst gamers.

However, it is important to note that structural similarities with gambling are not confined to loot boxes. As noted in Griffiths & Auer (2019), everyday activities such as fishing can also be seen to contain similar formal features to gambling. For example, both when fishing and when gambling, “the participant repeats the same behaviour over and over again in the hope that they will attain something of material value”.

Therefore, whilst formal similarities between loot boxes and gambling may be suggestive of a potential for adverse effects on gamers, they should not be treated as evidence either for or against real-world harm. In order to establish this evidence, empirical research is necessary. Thus far, such research is in its infancy. Correlational studies have shown that spending on loot boxes is linked to increases in problem gambling severity. The more money that gamers spend on loot boxes, the more severe their problem gambling tends to be. This effect appears reliable, and has been replicated in several studies (Zendle & Cairns, 2018; Zendle & Cairns, 2019; Zendle et al., 2019; Brooks & Clark, 2019; Zendle, Meyer & Over, 2019; Macey & Hamari, 2018). However, crucially, studies which report this effect have all been correlational in nature: they have involved simply surveying groups of gamers about both their problem gambling and their loot box spending and measuring the strength of links between these factors. It is therefore unclear what this link represents.

Loot box spending may be linked to problem gambling because loot boxes literally cause individuals to develop problem gambling, as suggested in Drummond & Sauer (2018). There are good theoretical reasons for believing that this may be the case. As noted in Blaszczynski & Nower (2002), a common step along a path to problem gambling is a process of conditioning in which intermittent rewards cause individuals to associate gambling activities with physiological excitement. This leads to increased participation in gambling, and the development of habitual patterns of gambling and attendant problems. Similarly, exposure to the intermittent wins that characterise loot boxes may result in a similar process of conditioning in which loot box spenders learn to associate gambling-like experiences with excitement. This may lead to engagement in gambling activities. Hence, exposure to loot boxes may lead to increased participation in gambling, and therefore increases in problem gambling amongst gamers.

Alternatively, loot box spending may be linked to problem gambling because individuals with pre-existing gambling problems are more likely to spend money on loot boxes. As noted in Zendle & Cairns, (2018), problem gambling is characterised by excessive and uncontrolled spending on gambling-related activities. Since loot boxes share many key characteristics with gambling, it is possible that disordered spending on gambling activities amongst problem gamblers transfers to loot boxes. Hence, loot box spending may be linked to problem gambling because individuals with pre-existing gambling problems may engage with a game that features loot boxes, be presented with gambling-like stimuli, and then spend heavily on them.

However, as noted in Zendle & Cairns (in press), a third possibility for the existence of this link exists. Problem gamblers often exhibit elevated levels of impulsivity (Michalczuk et al., 2011; Alessi & Petry, 2003). This may lead to overspending on a variety of in-game purchases, including loot boxes. In other words, loot box spending may not be linked to problem gambling because of unique gambling-like features of loot boxes but may instead represent a broader trend for overspending on all in-game microtransactions amongst problem gamblers.

In this study, we investigate which of these pathways is responsible for the observed links between loot box spending and problem gambling. We surveyed a sample of players of *Heroes of the Storm* (*n* = 112) both before and after the removal of loot boxes from the game. We measured how much these players spent in-game both before and after the removal of loot boxes, and the severity of their problem gambling. By doing so we established whether removing loot boxes from a game uniquely affected spending amongst problem gamblers, as opposed to other groups of gamers.

## Method

Ethical approval for this study was granted by York St. John University Ethics Committee (Approval ID: 2161). The data associated with this study is available at https://osf.io/vfw46/. Informed consent was obtained from participants prior to taking part in both waves of the study via an online form.

### Design

We conducted two online surveys with a sample of players of *Heroes of the Storm* aged 18 or older. The first of these surveys took place between March 21st and March 24th 2019 (**Time 1,** before the removal of loot boxes); the second survey took place between May 24th and June 3rd 2019 (**Time 2,** after the removal of loot boxes).

Participants were recruited via an advertisement on Amazon Mechanical Turk order to answer a survey about *Heroes of the Storm*.

### Screening questions

Participants were first screened to ensure that they played *Heroes of the Storm*. Participants were first asked, “During the last month, have you played Heroes of the Storm”. Any participants who answered ‘No’ to this question were not able to take part in the study.

Participants were then asked which of the following heroes they had used during the past month: Arthas Menethil, Grom Hellscream*, Chen Stormstout, Gelbin Mekkatorque*, and Malfurion Stormrage. The heroes that were marked with an * do not exist in *Heroes of the Storm*. Any participants who indicated that they had used one of these heroes during the past month were not able to take part in the study. At the end of the study, for the purposes of screening, they were asked these questions again to ensure consistency in their responses.

### Measured variables

**Problem gambling** was measured using the Problem Gambling Severity Index (PGSI) (Ferris & Wynne, 2001). The PGSI is a series of nine questions which measure how frequently individuals engage in behaviours that relate to problem gambling. Each of these questions is answered on a 4-point scale, with the following scoring pattern: (0) Never; (1) Sometimes; (2) Most of the time; (3) Almost always. The sum of scores over all 9 questions gives a total PGSI score that ranges from 0 (i.e., all questions answered as ‘Never’) to 27 (i.e., all questions answered as ‘Almost always’). Sample questions include “Thinking about the last 12 months, how often have you bet more than you could really afford to lose?” and “Thinking about the last 12 months, how often have you borrowed money or sold anything to get money to gamble?”. This scale showed a high degree of reliability (Cronbach’s alpha = 0.925).

Participants were then classified as either ‘non-problem gamblers’ (Score: 0), ‘low-risk gamblers’ (Score: 1–4), ‘moderate-risk gamblers’ (Score: 5–7), or ‘problem gamblers’ (Score: 8 +) using the revised scoring system for the PGSI (Currie, Hodgins & Casey, 2013).

Problem gambling was only measured at Time 1. Its questions involve ‘thinking about the last 12 months’ and therefore would not have shown significant variation at Time 2, which occurred only two months after Time 1.

**In-game spending** was measured by asking participants “Approximately how much money have you spent on microtransactions in Heroes of the Storm during the past month? Please give an estimate of this spending in US Dollars.”. This variable was measured at both Time 1 (before the removal of loot boxes) and at Time 2 (after the removal of loot boxes).

**Single session spending** was measured by asking participants “What is the *most money* you have you spent on microtransactions in Heroes of the Storm *in a single session* during the past month? Please give an estimate of this spending in US Dollars.”. This variable was measured at both Time 1 (before the removal of loot boxes) and at Time 2 (after the removal of loot boxes). It is important to note that single session spending was measured as a way of ensuring the reliability of in-game spending. Previous work on the effects of loot boxes has consistently used spending during the past month as an estimate of how much individuals spend on loot boxes (e.g., Zendle & Cairns, 2018; Zendle & Cairns, 2019; Zendle, Meyer & Over, 2019; Zendle et al., 2018). The intention was to replicate such analysis here. However, single session spending may in itself prove an interesting variable for study, as it may represent a degree of impulsivity in spending. The data associated with this study are available for use at the associated OSF repository, and reanalysis of this variable is possible. However, such analyses were not undertaken here.

Both **gender** and **age** were measured during Time 1. At the conclusion of Time 2, participants were asked “How has your experience of Heroes of the Storm changed since the game removed loot boxes?”. No further variables were measured during this study.

### Participants

A total of 371 responses were collected in total from players of *Heroes of the Storm* during Time 1. 23 respondents used duplicate IP addresses and were removed from the study. 91 respondents indicated that they spent more during a single session than they did during the whole month and were removed from the sample. Five respondents gave inconsistent answers to the screening questions when they were repeated at the end of the study and were removed from the sample. This left a total of 252 respondents for Time 1.

A total of 134 responses were collected from the recontact of valid Time 1 participants during Time 2. 2 respondents used duplicate IP addresses and were removed from the study. 20 respondents indicated that they spent more during a single session than they did during the whole month and were removed from the sample. This left a total of 112 participants overall.

Eighty participants described themselves as male. Twenty-seven participants described themselves as female. The remaining five participants gave other answers to questions asking regarding gender.

Twenty-two participants were aged 18–24. 39 were aged 25–29; 31 were aged 30–34; 11 were aged 35–39; nine were aged 40 or over.

It is interesting to describe the demographic details of individuals who were excluded from the study for indicating impossible levels of spending. The 91 participants at time 1 who were excluded from the study for this reason, on average, gave higher ratings of problem gambling severity than the rest of the sample: indeed, 58 of these participants were categorised as problem gamblers under their responses to the PGSI. This group may also have potentially been more likely to contain extreme responses: one individual in this group indicated that they had spent $30,000 on *Heroes of the Storm* in the past month. Indeed on average these individuals indicated that they spent more money on *Heroes of the Storm* than other individuals: mean spending in this group was $390.13 per month. However, this mean appears to be inflated by the presence of the outlier listed above: A 5% trimmed mean on spending was calculated at only $46.23. 38 of these participants listed themselves as female; 51 listed themselves as male; and two listed themselves as ‘f’ and ‘men’.

## Results

The effects of problem gambling severity and the presence of loot boxes on in-game spending were tested via a 4 × 2 mixed-design ANOVA, with time (before loot boxes were removed, after loot boxes were removed) as a within-participants factor and problem gambling severity (non-problem, low-risk, moderate-risk, problem gambler) as a between-participants factor.

**Table 1:** Paired *t*-tests showing the simple effects of time on in-game spending at each level of problem gambling severity.

**Problem gambling severity**	*t*	*df*	***p*-value**	**In-game spending before the removal of loot boxes**	**In-game spending after the removal of loot boxes**	**Mean difference (reduction in spending)**	**Cohen’s d**	Equivalent *η*^2^
Non-problem gamblers	0.033	58	1	$8.47($2.79–$14.15)	$8.47($3.41–$13.53)	$0.00	0	0
Low-risk gamblers	1.752	28	0.090	$11.37($5.54–$17.21)	$6.58($2.24–$10.93)	$4.79	0.325	0.025
Moderate-risk gamblers	−0.439	8	0.181	$21.11($6.63–$35.59)	$38.88($-0.15–$77.93)	-$17.77	0.488	0.056
Problem gamblers	2.420	14	0.029	$83.86($30.31–$137.41)	$48.46($0.15–$96.77)	$35.40	0.624	0.088

**Figure 1:**
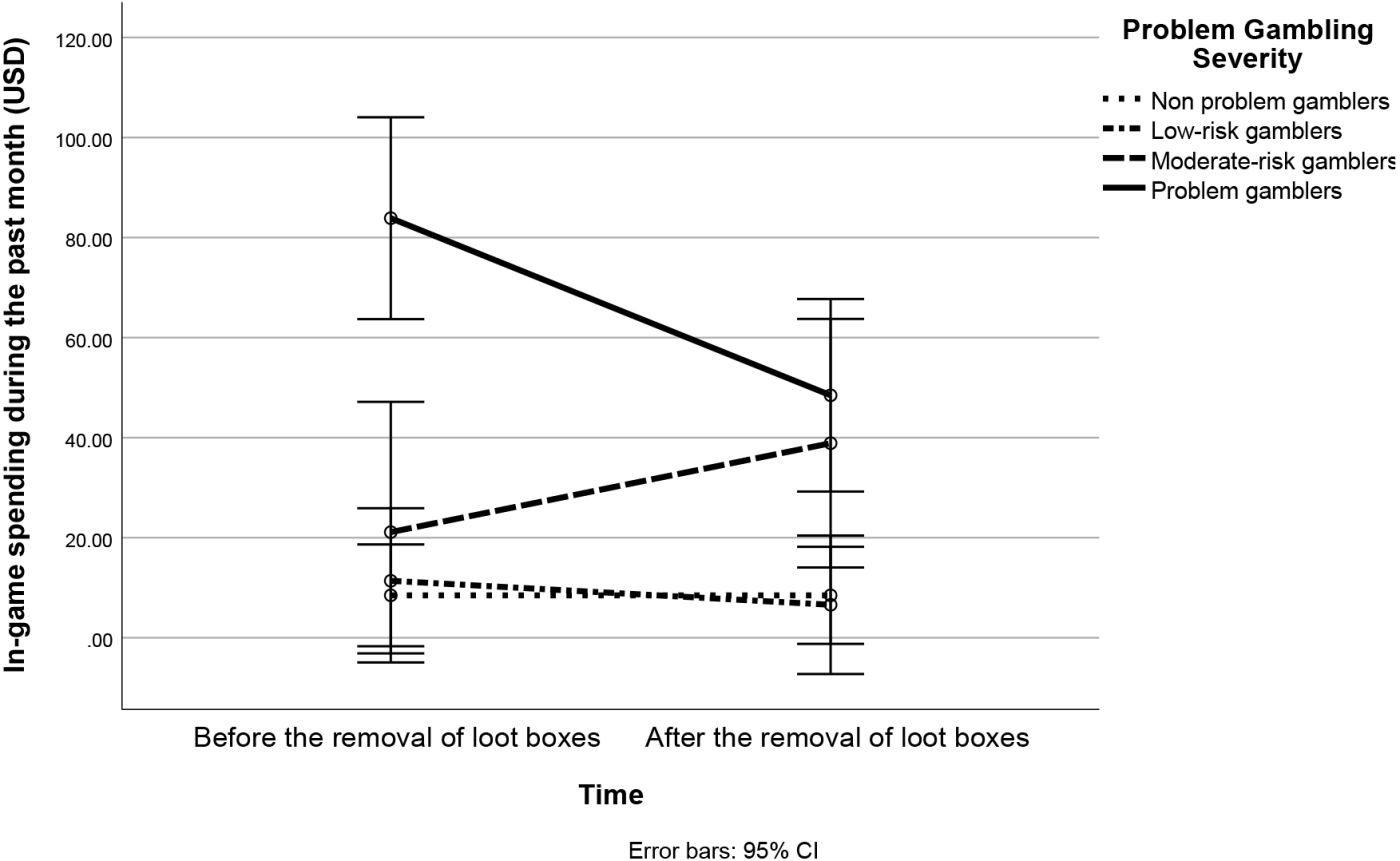
Line graph showing the interaction between time (before the removal of loot boxes, after the removal of loot boxes) and problem gambling severity (non-problem gamblers, low-risk gamblers, moderate-risk gamblers, problem gamblers).

Results indicated that there was no statistically significant main effect for time, F(1,108) = 2.973, *p* = 0.087, *η* ${}_{\mathrm{p}}^{2}=0.026$. There was a statistically significant effect for problem gambling severity F(3,108) = 11.33, *p* < 0.001, ${\eta }_{\mathrm{p}}^{2}=0.239$. There was a statistically significant interaction effect between time and problem gambling severity F(3,108) = 8.529, *p* < 0.001, ${\eta }_{\mathrm{p}}^{2}=0.191$. These results are depicted below as Fig. 1.

This interaction was clarified by examining the simple effects of time (before loot boxes were removed, after loot boxes were removed) on in-game spending at each level of problem gambling severity. This simple effects analysis was conducted via a series of paired-samples t-tests with time (before, after) a between-participants factor and in-game spending as a dependent variable. . Bonferroni corrections were applied for multiple comparisons, raising the alpha level of tests to 0.05/4, or 0.012. These analyses are reported below as Table 1.

In order to more severely test the effects outlined above, the same tests conducted above were repeated using an Aligned Rank Transform, equivalent to a nonparametric ANOVA (Wobbrock et al., 2011). Results indicated that there was a statistically significant main effect for time, F(1,108) = 40.049, *p* < 0.001, *η* ${}_{\mathrm{p}}^{2}=0.270$. There was a statistically significant effect for problem gambling severity F(3,108) = 11.485, *p* < 0.001, ${\eta }_{\mathrm{p}}^{2}=0.241$. There was a statistically significant interaction effect between time and problem gambling severity F(3,108) = 16.709, *p* < 0.001, ${\eta }_{\mathrm{p}}^{2}=0.317$.

The interaction effect was clarified by examining the simple effects of time (before loot boxes were removed, after loot boxes were removed) on in-game spending at each level of problem gambling severity. This simple effects analysis was conducted via a series of Wilcoxon tests with time (before, after) a between-participants factor and in-game spending as a dependent variable. Bonferroni corrections were applied for multiple comparisons, raising the alpha level of tests to 0.05/4, or 0.012. These analyses are reported below as Table 2.

### Exploratory analyses

#### Analyses of percentile decline

In order to better understand the relationship outlined above, decline in spending was reanalysed, not as an absolute dollar amount, but as a percent of spending at time 1. In order to calculate this, the following formula was used. In order to include cases where spending at time 1 was equal to $0, *ϵ* = 0.001 was included in both the numerator and denominator terms of the equation:

\begin{eqnarray*}Decline=100\ast \left( \frac{Spendin{g}_{t2}+\epsilon }{Spendin{g}_{t1}+\epsilon } \right) -100. \end{eqnarray*}

In order to determine whether there were significant differences in the percentile decline in spending between different groups of individuals of different problem gambling severities, a Kruskal-Wallis test by ranks was conducted, with problem gambling severity as the independent variable, and percentile decline in spending as the dependent variable. Results of the test indicated that there was a significant difference in percentile decline in spending between groups (*χ*^2^(3) = 10.078, *p* = 0.017).

Follow-up Mann Whitney U tests were conducted in order to clarify the nature of this difference. Results of these indicated that percentile decline was higher amongst problem gamblers than non-problem gamblers (*U* = 105.5, *p* = 0.025), and percentile decline was higher amongst problem gamblers than moderate risk gamblers (*U* = 604, *p* = 0.018). Additionally, percentile decline was significantly higher amongst moderate risk gamblers than amongst low risk gamblers (*U* = 75, *p* = 0.049).

**Table 2:** Nonparametric Wilcoxon tests showing the simple effects of time on in-game spending at each level of problem gambling severity. Effects significant at the *p* < 0.012 level are marked with an *.

**Problem gambling severity**	**Z**	*p*-value	**Cohen’s d**	Equivalent *η*^2^
Non-problem gamblers	0.653	0.513	0.170	0.007
Low-risk gamblers	2.078	0.037	0.836	0.148
Moderate-risk gamblers	1.266	0.205	0.931	0.178
Problem gamblers	2.629	0.008	1.848	0.460

No other significant differences were observed: there was no significant difference in decline between problem gamblers and low risk gamblers (*U* = 265.5, *p* = 0.223), no significant difference in decline between moderate risk gamblers and non-problem gamblers (*U* = 194, *p* = 0.157), and no significant difference in decline between low risk gamblers and non-problem gamblers (*U* = 1023.5, *p* = 0.095).

#### Analyses when impossible spenders are included

Several participants were not included in our analyses due to giving seemingly impossible information regarding their spending: for instance, claiming to spend more in a single session than they did in a whole month.

It is important to consider what our results might look like if these participants were included during analysis (*n* = 146). The Aligned Rank Transform nonparametric ANOVA was repeated with this larger sample. Results indicated that there was a statistically significant main effect for time, F(1,142) = 14.597, *p* < 0.001, *η*_p_^2^ = 0.235. There was a statistically significant effect for problem gambling severity F(3,142) = 23.458, *p* < 0.001, *η*_p_^2^ = 0.331. There was a statistically significant interaction effect between time and problem gambling severity F(3,142) = 5.456, *p* = 0.001, *η*_p_^2^ = 0.103.

The interaction effect was clarified by examining the simple effects of time (before loot boxes were removed, after loot boxes were removed) on in-game spending at each level of problem gambling severity. This analysis was conducted via a series of Wilcoxon tests with time (before, after) as a between-participants factor and in-game spending as a dependent variable. There was no significant change in spending observed amongst non-problem gamblers (*Z* = 0.978, *p* = 0.327). Low-risk gamblers spent significantly less after loot boxes were removed (*Z* = 2.450, *p* = 0.014). There was no significant change in spending observed amongst moderate-risk gamblers (*Z* = 0.769, *p* = 0.441). Problem gamblers spent significantly less after loot boxes were removed (*Z* = 2.187, *p* = 0.028).

## Discussion

A mixed design ANOVA showed a significant interaction between problem gambling severity and time (*p* < 0.001, ${\eta }_{\mathrm{p}}^{2}=0.191$).

However, despite the large magnitude of this interaction effect, initial follow-up subgroup analyses proved inconclusive. No significant reduction in spending was observed amongst either non-problem gamblers, low-risk gamblers, or moderate risk gamblers. A seemingly significant (*p* = 0.029) reduction in spending was observed amongst problem gamblers, with mean spending in this group dropping by a small to moderate amount (*d* = 0.624, equivalent to *η*^2^ = 0.088). However, when Bonferroni corrections were applied to the results of this test, raising the alpha level to 0.05/4, or 0.012, this result did not meet the criterion for statistical significance.

In order to clarify the nature of this effect, nonparametric analyses were undertaken. Nonparametric analyses are commonly used to analyse loot box spending due to the presence of extreme outliers in datasets (e.g., Zendle & Cairns, 2019; Zendle, Meyer & Over, 2019). These analyses painted a clearer picture. An Aligned Rank Transform (equivalent to a nonparametric mixed design ANOVA) again showed a significant interaction between problem gambling severity and time (*p* < 0.001).

Follow-up analyses to this test showed a clearer pattern of effects: in this case, a significant reduction in spending was seen amongst problem gamblers even when Bonferroni corrections were taken into account, *p* = 0.008. The magnitude of this effect was large: a Cohen’s d of 1.848, equivalent to *η*^2^ = 0.460. When Bonferroni corrections were applied to the results of analysis, no significant reduction in spending was observed amongst either non-problem gamblers, low-risk gamblers or moderate-risk gamblers. However, it is important to note that if these corrections were not applied, a significant reduction in spending would have been seen amongst low-risk gamblers (*p* = 0.037).

The analysis above defines an observed effect of magnitude *η*^2^ = 0.460. Effects that are larger in magnitude than *η*^2^ = 0.04 are commonly considered to bear real-world importance (Ferguson, 2009). This effect greatly exceeds that cut-off. However, if we look at the effect in a different light it may not appear so important: amongst problem gamblers, spending dropped from $ to $ when loot boxes were removed from a game. Is such a drop really worthy of attention? The answer to this is unclear –it

In order to further probe the nature of these effects, an exploratory analysis was undertaken which examined whether reductions in spending in percentile terms (rather than absolute dollar amounts) varied by problem gambling severity. Results indicated that there was a significant reduction in spending in percentile terms amongst problem gamblers. However, they also indicated a significant reduction in spending in percentile terms amongst low-risk gamblers. Finally, an exploratory analysis was conducted which examined what the results would resemble if individuals who were removed for specifying impossible patterns of spending were retained in the dataset. Problem gamblers still spent significantly less money when loot boxes were removed from *Heroes of the Storm*; however, under this analysis, low-risk gamblers also spent significantly less money in this situation.

Overall, therefore, these results paint a picture which suggests that when loot boxes are removed from a game, problem gamblers may uniquely spend less money on that game. However, the effects seen are somewhat inconsistent. For example, when Bonferroni corrections are applied to the data, a drop in spending amongst problem gamblers is only seen under nonparametric analysis. When Bonferroni corrections are not applied to the data, a drop in spending is also observed amongst low-risk gamblers. Similarly, when one analyses percentile reductions in spending, significant reductions appear amongst both low-risk and problem gamblers. Therefore, whilst these results might be suggestive of a situation in which problem gamblers—and only problem gamblers - spend less money when loot boxes are removed from a game, preregistered replication work is needed to confirm the reliability of this effect.

## Conclusions

Previous research has suggested that correlations between loot box spending and problem gambling may be due to three mechanisms. (1) They may occur because loot box spending causes problem gambling. (2) They may occur because the unique, gambling-like nature of loot boxes causes problem gamblers to spend more money on loot boxes in specific. (3) They may occur because of a general dysregulation amongst problem gamblers when it comes to spending in video games.

Our results do not support arguments that links between loot box spending and problem gambling are simply a consequence of general dysregulation in in-game spending amongst problem gamblers. When loot boxes were removed from *Heroes of the Storm*, problem gamblers spent significantly less money in-game. Furthermore, exploratory analyses revealed that there was a significant link between problem gambling severity and percentile reduction in spending when loot boxes were removed from a game: the more severe an individual’s problem gambling, the more their spending was reduced when loot boxes were removed from a game.

However, it is important to note that these results were somewhat inconsistent under some analyses. When Bonferroni corrections were applied to parametric analyses, the effect of removing loot boxes on spending amongst problem gamblers became statistically insignificant; when such corrections were *not* applied to the test, low-risk gamblers appeared to reduce their spending significantly when loot boxes were removed as well. It may be the case that the observed inconsistency is simply a product of the overtesting of a relatively small (*n* = 112) dataset. Further replication work is necessary to determine the reliability of the effects seen here.

When looking at the data presented here, a sceptic may note that whilst the effect size associated with the reduction in spending amongst problem gamblers was statistically large (*η*^2^ = 0.460), it only represented a real-world reduction in spending of $35.40. What, then, is the practical potential for financial harm in loot boxes? There are several possible rebuttals to such an observation. It is important to note that whilst the mean spending amongst problem gamblers may have dropped by only a small amount, individual problem gamblers themselves experienced a large drop in spending For example, if one consults the raw data associated with this study, one will observe individual problem gamblers whose spending decreased by hundreds of dollars when loot boxes were removed from *Heroes of the Storm*. Furthermore, on might argue that spending in general on *Heroes of the Storm* was relatively low for a video game. Even prior to the removal of loot boxes, problem gamblers were only spending, on average, $83.86 per month. The relationship seen here may take on a much larger real-world importance when viewed in the light of potentially more lucrative games, such as *FIFA* or *Clash Royale*. Significant further work using industry data on player spending is needed to fully answer these questions.

What are the real-world implications of this research? Problem gamblers are characterised by their disordered and excessive spending on gambling activities. This research may suggest that the presence of loot boxes in a game specifically causes problem gamblers to spend more money on that game. It seems possible that this is due to the gambling-like nature of loot boxes: as noted in Zendle & Cairns (2018), excessive and disordered spending on gambling activities may transfer to loot boxes due to formal similarities between themselves and gambling.

Loot boxes may have generated as much as $30 billion for the video game industry in 2018 alone (Juniper Research, 2018). These results suggest that this revenue may have been driven, at least in part, by gambling problems amongst gamers.

## Additional Information and Declarations

### Data Availability

The following information was supplied regarding data availability: Data is available at OSF: Zendle, David. 2019. “Supplemental Materials for Preprint: Only Problem Gamblers Spend Less Money When Loot Boxes Are Removed from a Game: A before and after Study of Heroes of the Storm.” OSF. July 24. https://osf.io/vfw46/.
